# Astroglia-Microglia Cross Talk during Neurodegeneration in the Rat Hippocampus

**DOI:** 10.1155/2015/102419

**Published:** 2015-04-21

**Authors:** Montserrat Batlle, Lorenzo Ferri, Carmen Andrade, Francisco-Javier Ortega, Jose M. Vidal-Taboada, Marco Pugliese, Nicole Mahy, Manuel J. Rodríguez

**Affiliations:** ^1^Unitat de Bioquímica i Biologia Molecular, Facultat de Medicina, Institut d'Investigacions Biomèdiques August Pi i Sunyer (IDIBAPS), Universitat de Barcelona and Centro de Investigación Biomédica en Red Sobre Enfermedades Neurodegenerativas (CIBERNED), C/Casanova 143, 08036 Barcelona, Spain; ^2^Istituto di Anatomia Umana e Biologia Cellulare, Facoltà di Medicina e Chirurgia, Università Cattolica del Sacro Cuore, 00168 Roma, Italy; ^3^Vall d'Hebron Institute of Research (VHIR), Passeig de la Vall d'Hebron 119, 08035 Barcelona, Spain

## Abstract

Brain injury triggers a progressive inflammatory response supported by a dynamic astroglia-microglia interplay. We investigated the progressive chronic features of the astroglia-microglia cross talk in the perspective of neuronal effects in a rat model of hippocampal excitotoxic injury. N-Methyl-D-aspartate (NMDA) injection triggered a process characterized within 38 days by atrophy, neuronal loss, and fast astroglia-mediated S100B increase. Microglia reaction varied with the lesion progression. It presented a peak of tumor necrosis factor-*α* (TNF-*α*) secretion at one day after the lesion, and a transient YM1 secretion within the first three days. Microglial glucocorticoid receptor expression increased up to day 5, before returning progressively to sham values. To further investigate the astroglia role in the microglia reaction, we performed concomitant transient astroglia ablation with L-*α*-aminoadipate and NMDA-induced lesion. We observed a striking maintenance of neuronal death associated with enhanced microglial reaction and proliferation, increased YM1 concentration, and decreased TNF-*α* secretion and glucocorticoid receptor expression. S100B reactivity only increased after astroglia recovery. Our results argue for an initial neuroprotective microglial reaction, with a direct astroglial control of the microglial cytotoxic response. We propose the recovery of the astroglia-microglia cross talk as a tissue priority conducted to ensure a proper cellular coordination that retails brain damage.

## 1. Introduction

Injury to the central nervous system, including stroke and traumatic brain injury, induces excitotoxic neuronal death that triggers a potent inflammatory response with a dynamic astroglia and microglia reaction that determine neuronal fate [1, 2]. Following the injury, all cells present a metabolic reprogramming to cover the bioenergetic and substrate demand for the trophic/inflammatory processes to take place [3] with the coexistence of various factors.

An excessive production of proinflammatory factors from activated microglia, such as tumor necrosis factor-*α* (TNF-*α*), interleukin-1*β*, and reactive oxygen species, may trigger or exacerbate neuronal death [4]. Microglia do not constitute a unique cell population and show a range of phenotypes that are closely related with the evolution of the damaging process [5, 6]. Thus, their control will directly influence the tissue outcome [7]. These phenotypes range from the well-known proinflammatory activation state to a trophic one involved in cell repair and extracellular matrix remodelling [8]. In neurodegeneration, for example, microglia show inflammatory and neuroprotective properties [6] associated with the expression of YM1, a secretory protein related to neuroregeneration [9, 10]. In addition, as some microglial cells become increasingly dysfunctional, they may directly participate in the development of neurodegeneration [11].

Whether microglia through TNF-*α*, interleukin-1*β*, and reactive oxygen species formation [4] adopt a phenotype that mostly exacerbates tissue injury or promotes brain repair with YM1 and other neuroprotective factors expression [9, 10] likely depends on the diversity of signals from the lesion environment, especially at the quadripartite synapse level [12]. For instance, astroglial chemokines have an influence upon microglia/macrophage activation in multiple sclerosis with CCL2 (MCP-1) and CXCL10 (IP-10) directing reactive gliosis [13]. Although a clear account of this dynamic relationship has yet to be proposed, the astrocyte-microglia interplay might determine the phenotype that microglia adopt during neurodegeneration.

Steroid hormones may modulate the microglial response to injury, although the results are still controversial. Studies have shown that glucocorticoids regulate peripheral immune responses and have CNS anti-inflammatory properties, but they also appear to develop proinflammatory effects that exacerbate excitotoxicity and cerebral damage (see [14] for a review). This observed variability may be related to an acute or chronic CNS effect, with the initial anti-inflammatory modulation being eventually followed by an exacerbation of cerebral injury [14]. If this hypothesis is correct, it would be possible to modulate the cytotoxic and neuroprotective activity of microglia through acute or chronic activation of the glucocorticoid receptor (GR), the most abundant steroid hormone receptor found in microglia [15].

Astroglial S100B is one of those factors that control microglial activity. Astrocytes release S100B constitutively [16] and increase this release upon stimulation by several factors, including TNF-*α* [17]. Under normal conditions, released S100B acts as a neurotrophic factor, countering the stimulatory effect of neurotoxins on microglia [18] and stimulating glutamate uptake [19]. By contrast, at high concentrations S100B binds the Receptor for Advanced Glycation End products (RAGE), which might mediate microglial activation in the course of brain damage [20]. Thus, secreted S100B participates in astrocyte-microglia cross talk, with an important role in the initial phase of brain insults.

The purpose of the present study was to study the relationship between microgliosis and astrogliosis in an* in vivo* model of hippocampal injury that triggers chronic neuroinflammation [12]. To this end, we undertook a time-course study between day 1 and day 38 of a hippocampal N-methyl-D-aspartate (NMDA) stereotaxic-induced lesion and we characterized neuronal loss and the astroglial and microglial reactions. We quantified TNF-*α*, YM1, 8 kDa translocator protein (TSPO, or peripheral benzodiazepine receptor, PBR [21]), and GR at different postlesion times to estimate the state of microglial activity. Then, to determine the relationship between astrogliosis and microglia activation, we coinjected NMDA with L-*α*-aminoadipate (*α*-AA), a specific astrotoxin used to investigate astroglial participation in several paradigms (e.g., [22–25]). *α*-AA induces a transient astroglial ablation, which is associated with a microglial reaction that persists over several days [24, 26, 27]. The sequence of events described* in vivo* after *α*-AA stereotaxic microinjection includes astroglial degeneration for 1–3 days, microglial invasion, and, finally, astroglial recovery [24, 26].

## 2. Materials and Methods

### 2.1. Animals

Adult male Wistar rats (body weight 200–225 g at the beginning of the study) were obtained from the animal housing facilities at the School of Medicine (Universitat de Barcelona). They were kept on a 12 h/12 h day/night cycle and housed with free access to food and water. Animals were manipulated according to European legislation (86/609/EEC) and all efforts were made to minimize the number of animals used and their suffering. The number of animals to be included in each group was statistically estimated to be 6 rats/group (tolerance interval ±0.9, confidence level 95%). Procedures were approved by the Ethics Committee of the Universitat de Barcelona, in accordance with the regulations established by the Catalan government (Generalitat de Catalunya).

### 2.2. Chemicals

NMDA, *α*-AA, the mouse monoclonal anti-glial fibrillary acidic protein (GFAP), and the biotin-conjugated isolectin B4 (IB4) from* Bandeiraea simplicifolia* were all purchased from Sigma (St. Louis, MO). The mouse monoclonal anti-NeuN was purchased from Chemicon (Temecula, CA), the mouse anti-rat CD11b antibody (clone MRC OX-42) was from Serotec Ltd. (Oxford, UK), and the rabbit polyclonal anti-S100B antibody was from DAKO (Dako Diagnostics, Barcelona, Spain). The goat polyclonal AMCase (M-19) antibody that specifically binds the transcription factor YM1 was from Santa Cruz Biotechnology (Santa Cruz, CA) as was the rabbit polyclonal anti-GR antibody. Secondary antibodies and immunohistochemical reagents were from Sigma.

[^3^H]PK-11195 was purchased from Perkin-Elmer (Boston, MA). [^3^H]Corticosterone was from Amersham Bioscience (Bucks, UK) and RU-28362 was purchased from Sigma. Dodecyl sulphate-polyacrylamide gel electrophoresis standards were purchased from Bio-Rad (Hercules CA), Immobilon-P membranes were from Millipore (Bedford, MA), and ECL Plus Western Blotting reagent was from Amersham Bioscience (Bucks, UK). The murine TNF-*α* ELISA development kit was purchased from PeproTech (Paris, France).

### 2.3. Stereotaxic Procedure and Labeling of Proliferating Cells

Under equithesin anesthesia (a mixture of chloral hydrate and sodium pentobarbitone; 0.3 mL/100 g body wt, i.p.), rats were placed in a stereotaxic instrument (David Kopf, Carnegie Medicin, Sweden) with the incisor bar set at −3.3 mm. According to the Atlas of Paxinos and Watson [28], the stereotaxic coordinates for the hippocampal microinjection were 3.3 mm caudal to bregma, 2.2 mm lateral to bregma, and 2.9 mm ventral from dura. A 5.0 *μ*L Hamilton syringe activated by an infusion pump (CMA/100; Carnegie Medicin, Sweden) was used for the intracerebral injection into the hippocampal parenchyma. In all injections 0.5 *μ*L was infused over 5 min as previously described [29].

Animals received a single injection of either 50 mM phosphate buffer saline (PBS; pH 7.4) (sham group), 20 nmol NMDA in 50 mM PBS (NMDA group), 6.4 nmol *α*-AA (*α*-AA group), or 20 nmol NMDA plus 6.4 nmol *α*-AA (NMDA + *α*-AA group). At five different postlesion times (1, 3, 5, 15, and 38 days) a total of 60 rats, 12 for each group (sham, *α*-AA, NMDA, and *α*-AA + NMDA) and 12 control rats (with no treatment, assigned as 0 days), were anaesthetized and decapitated. Half of the animals (6 rats/group) were used for biochemical studies and the other half were used for histological approaches. The NMDA dose was chosen according to previous studies [29]. As it was used in low concentrations, sodium pentobarbitone did not interfere with the function of NMDA receptors [29]. *α*-AA was at the limit of its solubility in water (2.2 mg/mL) and the selected dose ensured a specific gliotoxic effect [24].

### 2.4. Histology, Immunohistochemistry, and Image Analysis

At the indicated postlesion time, six rats from each group were anaesthetized and decapitated. Their brains were then quickly removed, frozen with powdered dry ice, and stored at −80°C until use. Adjacent 14 *μ*m coronal serial sections at the level of the dorsal hippocampus (−3.3 mm to bregma) were obtained from all brains, mounted on slices, and processed for histology, immunohistochemistry, and* in vitro* autoradiography studies.

Standard Nissl staining was performed to evaluate neuronal loss and the morphology of the hippocampal region. Microglial cell shapes were identified by histochemistry with biotin-conjugated IB4 [30]. Briefly, endogenous peroxidase activity was inhibited by a 10-minute preincubation in H_2_O_2_-methanol-PBS (0.3/9.7/90) followed by a 10-minute wash in PBS [31] and postfixation for 10 min with ice-cold paraformaldehyde (4% in PBS, pH 7.4). Then sections were incubated overnight at 4°C with IB4 diluted 1 : 25 in normal goat serum (1 : 100 v/v in 0.01 M PBS; pH 7.4). After incubation with ExtrAvidin (1 : 250), sections were developed in a 0.05 M Tris solution containing 0.03% (w/v) diaminobenzidine and 0.006% (v/v) H_2_O_2_.

Immunohistochemistry was carried out by the biotin-avidin-peroxidase method. Astroglial reaction was assessed by immunodetection with mouse monoclonal anti-GFAP and rabbit polyclonal anti-S100B antibodies diluted 1 : 400 and 1 : 800, respectively. Neuronal staining and GR expression were evaluated, respectively, with mouse monoclonal anti-NeuN antibody (1 : 150) and rabbit polyclonal anti-GR antibody (1 : 750). In brief, endogenous peroxidase activity was inhibited by a 10-minute preincubation in H_2_O_2_-methanol-PBS (0.3/9.7/90) followed by a 10-minute wash in PBS. Then, after postfixation for 10 min with ice-cold paraformaldehyde (4% in PBS, pH 7.4), sections were incubated overnight at 4°C with the primary antibody at the appropriate dilution in 0.05 M PBS containing 0.5% Triton ×100, 1% normal goat serum, and 1% bovine serum albumin. After washing and incubating with the appropriate secondary antibody, sections were incubated with ExtrAvidin (1 : 250) and developed in diaminobenzidine and H_2_O_2_.

Double immunohistochemistry with specific cellular markers was performed to determine the cells expressing glucocorticoid receptors. Sections were coincubated overnight at 4°C with the rabbit polyclonal anti-GR antibody (1 : 750) and either mouse monoclonal anti-NeuN antibody (1 : 250) for neurons, mouse monoclonal anti-GFAP antibody (1 : 750) for astroglial cells, or mouse monoclonal anti-CD11b (1 : 100) for microglia. After washing, sections were sequentially incubated in the dark with goat anti-mouse IgG Alexa Fluor-555 conjugated (1 : 300) to detect cellular phenotype and then with biotinylated goat anti-rabbit IgG (1 : 200) followed by FITC-conjugated ExtrAvidin (1 : 250) to detect GR.

All double immunostained sections were mounted in ProLong antifade and kept in the dark. Incubations with either mouse or goat IgG as primary antibodies were used for negative controls. Confocal images were acquired using a Leica TCS SL laser scanning confocal spectral microscope (Leica Microsystems Heidelberg GmbH, Manheim, Germany).

Morphological and histological parameters were measured in 14 *μ*m thick serial coronal sections with the optical microscope software AxioVision 4 AC (Zeiss) and analyzed by the Image Pro Plus v.5.1 image analysis system (Media Cybernetics Inc., Bethesda, MD, USA). The hippocampal formation (HF) size, the area occupied by the neuronal loss, and the different hippocampal subfield areas were measured on Nissl stained sections at the injection level that allows differentiating between structures and identifying the damage to be accurately quantified. The area of hippocampus occupied by reactive astroglia or microglia was measured by delimitation of the increased immunoreactivity region on adjacent GFAP-immunostained, S100B-immunostained, and IB4-stained sections, respectively, using the same system [24]. Area size determinations were performed in three different stained sections at the injection level of all rat brains. To minimize biased errors due to fronto-occipital hippocampal size variations, only those sections located near the injection site and at similar bregma level were selected for quantification. Then, the area of interest was referred to as the whole HP area measured in each of the GFAP-immunostained, S100B-immunostained, and IB4-stained sections analyzed. Measurement of the whole HP area was also used to calculate the ratio between total HF and the area of gliosis. In all cases the contralateral hippocampus was measured in the same sections in order to estimate the effects of histological procedures on tissue size and thus to correct for variability in individual brain size and tissue shrinkage [32]. GFAP/S100B expression areas were estimated by the quotient of the increased immunoreactivity region in GFAP- and S100B-immunostained sections of each rat.

Neuronal cell counting was performed in NeuN immunostained sections. Under the optical microscope, we randomly selected four areas of interest (1.0 mm^2^ each) from the dorsal hippocampus. Inside of these areas, we counted the number of stained cells at a ×40 objective magnification. Cell counting was performed in duplicate in three different stained sections of all rat brains. Immunopositive cells were counted in all lesioned hippocampal subfields, and quantification was made in the Image Pro Plus v.5.1 image and analysis system. To allow for quantification of double immunostained cells, multiple epifluorescent partial images of each hippocampus were taken and mounted in order to reconstruct an image of the whole structure using a Leica DMI 6000B inverted microscope equipped with the Tile Scan function of the LAS AF Leica software (Leica Microsystems Heidelberg GmbH, Manheim, Germany). Counting of double-immunostained cells was performed in duplicate in three different stained sections of all rat brains as explained above. GR density was analysed densitometrically in bright field microscopy images taken from three adjacent GR immunostained sections [33] from the lesioned hippocampus. To do that, we used the same procedure and image analysis system as described above for NeuN immunopositive cells. The size and shape of the cell soma were used to discriminate between neurons and glial cells [33] (Figure 2(a)). For validation of this counting, the number of double immunopositive cells was also calculated in NeuN-GR and CD11b-GR double stained sections as explained above for the estimation of the number of proliferating cells.

### 2.5. *In Vitro* Autoradiography

Adjacent sections were processed for* in vitro* autoradiography to assess the hippocampal distribution of TSPO and GR. TSPO was labeled with [^3^H]PK-11195 as a microglial marker. Tissue sections were incubated for 2 h at room temperature in 50 mM Tris-HCl (pH 7.7) containing 1 nM [^3^H]PK-11195 (85 Ci/mmol). Nonspecific binding was determined in the presence of 1 *μ*M PK-11195. It was homogeneous and lower than 10% of the total binding. Glucocorticoid receptors were labelled with 10 nM [^3^H]corticosterone (79 Ci/mmol) added to a buffer solution containing 20 mM Tris-HCl (pH 7.4), 1.5 mM EDTA, 140 mM NaCl, and 5 mM glucose. 5 *μ*M RU-28362 was used to discriminate between GR and mineralocorticoid receptor specific [^3^H]corticosterone binding [34]. The nonspecific binding was determined by incubation with 20 *μ*M corticosterone and was lower than 20% of total binding.

After washing in the appropriate buffer, slides were dried overnight under a stream of air at 4°C and opposed to Hyperfilm-^3^H (Amersham) for a period between two weeks and two months. Films were developed and analysed densitometrically after calibration with plastic standards (^3^H-Microscales, Amersham) using the Image Pro Plus v.5.1 image analysis system. The average brain protein content was 8%. For each brain, four sections were processed for total binding and two other sections for nonspecific binding.

### 2.6. Western Blot and Immunoassay

At the indicated postlesion time, six rats from each group were anaesthetized and decapitated. The brain of each animal was removed and the HF dissected, before being quickly frozen in liquid N_2_ and stored at −80°C prior to use. Each HF was manually homogenized in ice-cold Tris-HCl 50 mM, pH 7.7, containing 2 mM EDTA and a protease inhibitor cocktail. Part of the homogenate was immediately ultrasonicated at 4°C with a Sonifier 250 (Branson, Ultrasonic Corp., Danbury, CT) and centrifuged; the supernatant was directly used for TNF-*α* immunoassay. The other part was ultracentrifuged at 15000 rpm for 15 min at 4°C and the supernatant (cytosolic fraction) was used for Western blot analysis of YM1 protein. In both cases, protein determination was carried out using the Bradford method.

Western blot analysis was performed as described elsewhere, with specific antibodies against YM1 [10]. Low-range molecular weight biotinylated SDS-PAGE standards were run in each gel to ascertain the position of each band. Immobilon-P was used for electroblotting and the immunocomplexes were detected by enhanced chemiluminescence using the ECL Plus Western Blotting Kit. Films were then developed, scanned, and analysed densitometrically with the Image Pro Plus v.5.1 image analysis system.

TNF-*α* was quantified with the murine TNF-*α* ELISA development kit as indicated by the supplier protocol. In all samples, TNF-*α* determination was performed in duplicate.

### 2.7. Statistical Analysis

For each parameter, kurtosis and skewness moments were calculated to test the normal distribution of data. A two-way ANOVA was performed with two factors: time with six levels (0 days, 1 day, 3 days, 5 days, 15 days, and 38 days) and treatment with four levels (sham, *α*-AA, NMDA, and NMDA + *α*-AA). When significant two-way interactions were observed, individual comparisons were performed using one-way ANOVAs followed by the LSD* post hoc* test. Single comparisons between sham and NMDA groups at one time point were made using the Student's *t*-test (*t*). When normality was not achieved, the values of all groups were compared using the nonparametric Kruskal-Wallis test (KW) followed by the Mann-Whitney *U* test (KS). In all cases, *P* < 0.05 was considered as statistically significant. Results are expressed as mean ± SEM. All analyses were performed with the STATGRAPHICS software (STSC Inc., Rockville, MD, USA).

## 3. Results

### 3.1. NMDA Induced Fast, Enduring Microgliosis with Phenotype Changes within Fifteen Days

**Figure 1:**
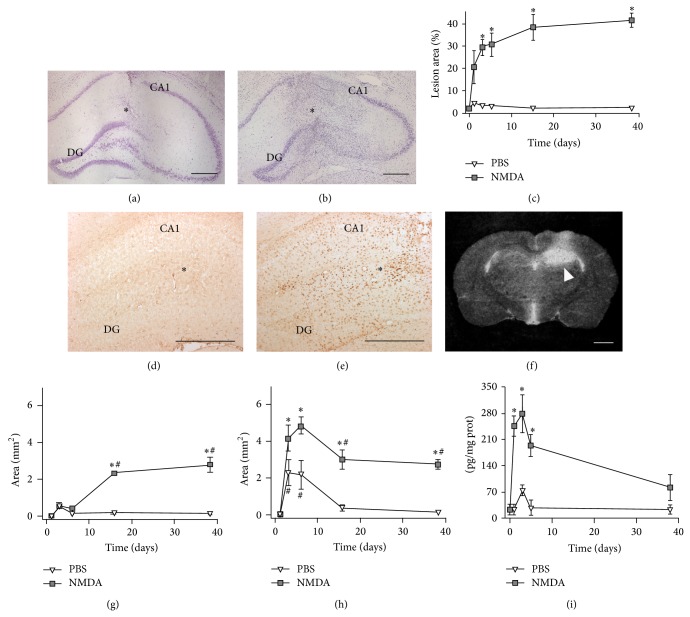
Timing of the microglial reaction to NMDA in the hippocampus. Photomicrographs illustrate the hippocampal injury and microglial reaction to NMDA-induced lesion at the injection site. Photomicrographs of cresyl-violet stained brain sections of (a) sham rats and (b) NMDA rats 15 days after the lesion. (c) Graph shows the quantification of the area of lesion relative to the whole HF in cresyl-violet stained sections. IB4 histochemistry of sham (PBS) (d) and NMDA rats (e) 15 days after the lesion. (f) Distribution of specific binding sites for [^3^H]PK-11195 in a coronal rat brain section 15 days after 20 nmol NMDA injection. Note the NMDA-induced increase of specific binding (arrowhead) seen as a white area in the left hippocampus. Graphs show the quantification of the area of microglial reaction in IB4-stained sections (g), the area of enhanced [^3^H]PK-11195 specific binding (h), and the hippocampal concentration of TNF-*α* (i) of PBS and NMDA rats during the 38 days of the study. Asterisks in (a), (b), (d), and (e) indicate the injection site. CA1,* Cornu Ammonis* area 1; DG, dentate gyrus. ^∗^ *P* < 0.05 different from PBS; ^#^ *P* < 0.05 different from day 0 (LSD* post hoc* test in (d), (f); KW test in (e)) (*n* = 6 PBS; *n* = 6 NMDA rats). Bar: 1 mm in (a), (b), (d), and (e) and 2 mm in (f).

Observation of Nissl-stained sections revealed that 20 nmol NMDA produced major layer disorganization, neuronal loss, and gliosis in all layers of the hippocampal formation (HF). Animals from the sham group showed no cellular alterations except for the needle scar (Figure 1(a)). The hippocampal lesion extended within 2 mm around the injection site in the rostrocaudal axis, with a slight tendency to grow towards the caudal direction. In the NMDA group (Figure 1(b)), we observed an increase in the area of lesion as a consequence of an initial massive neuronal loss within the first 3 days, followed by a more discrete neuronal death that still progressed at 38 days (Figure 1(c)).

Histochemistry analysis with IB4 stained hyperplasic and hypertrophic microglia in all NMDA-lesioned animals (Figure 1(e)). The reactive microglia area increased with time and reached a maximal value at 15 days, with this value being maintained at day 38 (Figure 1(g)). Quantification of the TSPO microglial expression by [^3^H]PK-11195 using* in vitro* autoradiography (Figure 1(f)) also showed an area of microglial reaction to the NMDA injection into the hippocampus (Table 1). In the NMDA-lesioned versus the sham groups both the intensity (Table 1) and the area (Figure 1(f)) of [^3^H]PK-11195 binding density showed an increase between day 1 and day 38 (Figure 1(h)). The sham group showed [^3^H]PK-11195 specific binding that was increased in the injection site only in a small area on day 1.

The TNF-*α* concentration increased in both sham and NMDA-lesioned groups only within the first five days of the lesion (Figure 1(i)). Between days 1 and 3, the concentration of TNF-*α* in the NMDA group increased with respect to the sham group and progressively returned to initial values by day 38. In the sham group, we only found a small transient increase in TNF-*α* concentration at day 3.

**Figure 2:**
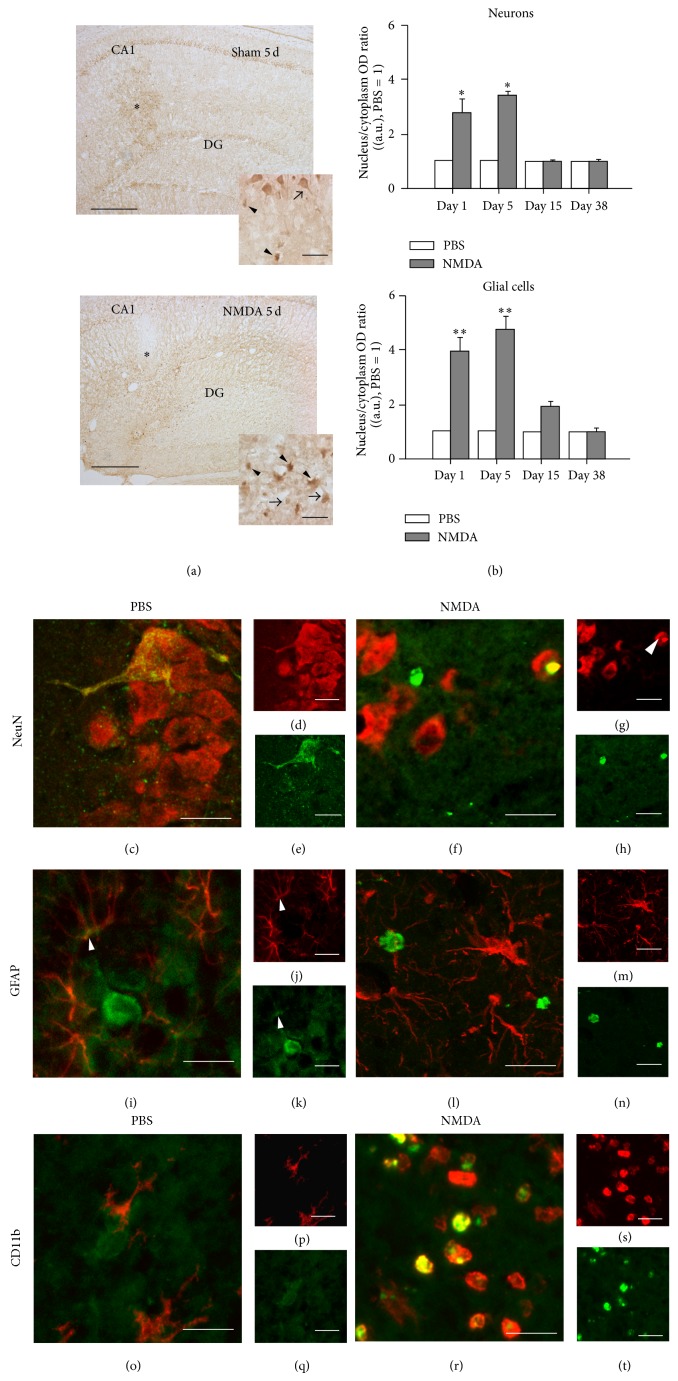
Activation of glucocorticoid receptors (GR) induced by NMDA injection in the hippocampus. (a) Photomicrographs illustrate GR immunohistochemistry in the hippocampus of sham animals and NMDA rats 5 days after the injection. Asterisks indicate the injection site and insets illustrate the GR immunostaining in neurons (arrow) and glial cells (arrowheads). Please note that pyramidal cells (arrows) are much bigger than glia (arrowheads); they also present a pyramidal shape with a dendritic tree, while reactive microglia have a round smaller shape. (b) Graphs show the quantification of the nucleus/cytoplasm ratio of GR-immunolabeling in hippocampal neurons and glial cells of sham (PBS) and NMDA-lesioned rats. Confocal photomicrographs show the double immunohistochemistry of GR (in green) with specific cellular markers (in red) in the hippocampal CA1 layer of NMDA-lesioned rats 5 days after the lesion. NeuN-GR double immunostaining of PBS (c–e) and NMDA (f–h) rats evidences an increased GR-immunolabeling in the nucleus of neurons of NMDA rats (arrowheads). GFAP-GR double immunostaining in PBS (i–k, arrowheads) and NMDA (l–n) rats shows an increased GFAP-immunolabeling in astrocytes of NMDA rats not associated with GR-immunoreactivity changes, which indicates that the NMDA-induced GR activation quantified in panel (b) is not astroglial. CD11b-GR double immunohistochemistry in PBS (o–q) and NMDA (r–t) rats evidences an increased GR-immunolabeling in the nucleus of reactive microglia of NMDA rats. ^∗^ *P* < 0.05; ^∗∗^ *P* < 0.01 different from PBS (Student's *t*-test) (*n* = 6 rats/group). Bar: 500 *μ*m in (a) and 10 *μ*m in (c)–(t) and insets in (a).

We quantified the time-related changes of glucocorticoid receptor (GR) concentrations in the hippocampus by immunohistochemistry (Figure 2) and by* in vitro* autoradiography of [^3^H]corticosterone binding (Table 1). In the sham group, single immunohistochemistry of GR showed widespread staining in the hippocampus and adjacent areas. We observed an increased GR-immunoreactivity (GR-IR) in the lesioned HF mostly in the nucleus of glial cells and surviving neurons between days 1 and 5 after NMDA injection (Figures 2(a) and 2(b)). Double immunohistochemistry and confocal analysis of anti-GR antibody with either anti-NeuN, anti-GFAP, or anti-CD11b antibodies evidenced short-term GR distribution changes in surviving neurons and reactive microglia but not in reactive astrocytes (Figures 2(c)–2(t)). We found the more evident GR-IR increase in the nucleus of round-shaped CD11b-immunostained cells at day 5 after the lesion (Figures 2(o)–2(t)), whereas the weak GR labeling of most GFAP-immunopositive cells remained and only a few scattered cells showed increased nuclear staining (Figures 2(i)–2(n)).

We calculated the nucleus/cytoplasm GR-IR ratio as an estimation of GR activation, as unactivated GR predominantly localized within the cytoplasm and migrates to the nucleus with hormone binding. At day 1 the nucleus/cytoplasm GR-IR ratio was strongly increased in neurons of the lesioned CA1 strata and remained increased only at day 5. We found marked differences in microglial cells (Figure 2(b)). In these cells, the nucleus/cytoplasm GR-IR ratio increased at days 1 and 5 after NMDA microinjection to then gradually decrease to sham group values at day 38.

Experiments with [^3^H]corticosterone specific binding to GR showed low levels in the HF and the parietal and pyriform cortex of sham animals (Table 1). In the hippocampus of NMDA-lesioned rats, [^3^H]corticosterone specific binding progressively increased reaching a maximal value of 448 ± 247 fmol/mg prot at day 5 to then decrease to sham levels at day 15 (Table 1).

### 3.2. Within Fifteen Days *α*-AA Did Not Modify NMDA-Mediated Neuronal Loss

**Figure 3:**
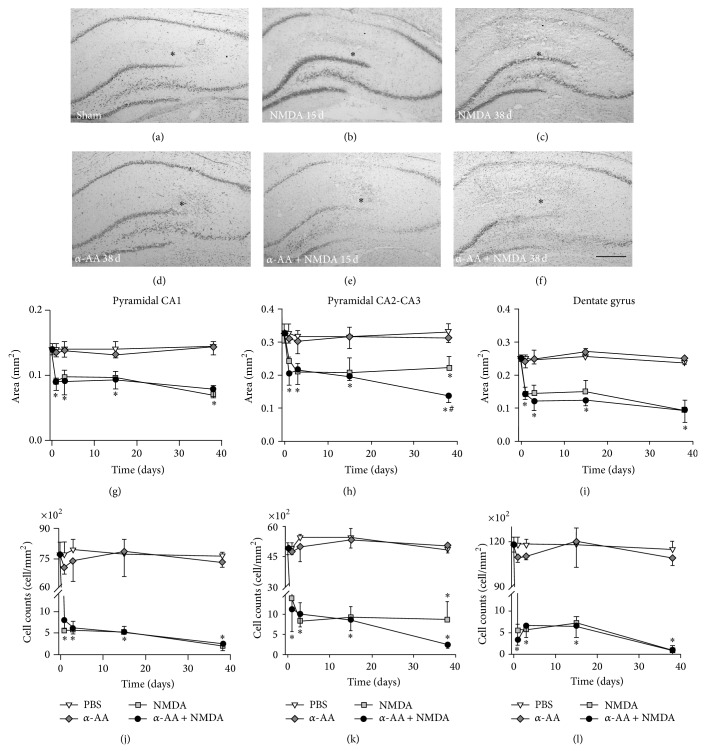
*α*-AA effect on the NMDA-induced hippocampal lesion. Photomicrographs of cresyl-violet stained brain sections of sham rats. (a) NMDA rats 15 days (b) and 38 days after the lesion (c). *α*-AA rats (d) and *α*-AA + NMDA rats 15 days (e) and 38 days after the lesion (f). Asterisks indicate the injection site. Graphs (g) to (i) show the quantification of the area of neuronal loss in cresyl-violet stained sections in the pyramidal CA1 (g), pyramidal CA2-CA3 (h), and dorsal dentate gyrus (i) of sham (PBS), *α*-AA, NMDA, and *α*-AA + NMDA rats at postlesion days 1, 3, 15, and 38. Graphs (j) to (l) show the estimation of the density NeuN immunopositive neurons in the pyramidal CA1 (j), pyramidal CA2-CA3 (k), and dorsal dentate gyrus (l) of the same rats. ^∗^ *P* < 0.05 different from PBS; ^#^ *P* < 0.05 different from NMDA (LSD* post hoc* test) (*n* = 6 rats/group). Bar: 500 *μ*m.

Stereotaxic Injection of 20 nmol NMDA produced major neuronal loss mainly in the CA1 layers (Figures 3(a)–3(c)). The area of neuronal loss extended to 34 ± 5% of the pyramidal CA1 within the first three days (Figure 3(g)) and then progressively increased to reach a maximal value at day 38. The same pattern was observed in the neuronal density measured in NeuN immunostained sections (Figure 3(j)).

When we coinjected 6.4 nmol *α*-AA with 20 nmol NMDA (*α*-AA + NMDA rats) all the lesion parameters were similar in all hippocampal strata of the NMDA and *α*-AA + NMDA groups during the first five days (Figures 3(d)–3(f)). In the *α*-AA + NMDA group, we observed increased tissue disorganization at day 15 and this area of lesion reached a maximal value at day 38 (Figures 3(d)–3(f)). At all time points, neuronal density in the HF of *α*-AA + NMDA and NMDA groups was similar (Figures 3(j)–3(l)).

The injection of 6.4 nmol of *α*AA alone caused no change in the hippocampal size, lesion area, or neuronal density when compared with sham group values at any of the studied time points (Figures 3(g)–3(i)).

### 3.3. *α*-AA Enhanced the S100B/GFAP Ratio Three Days after the NMDA-Induced Lesion

**Figure 4:**
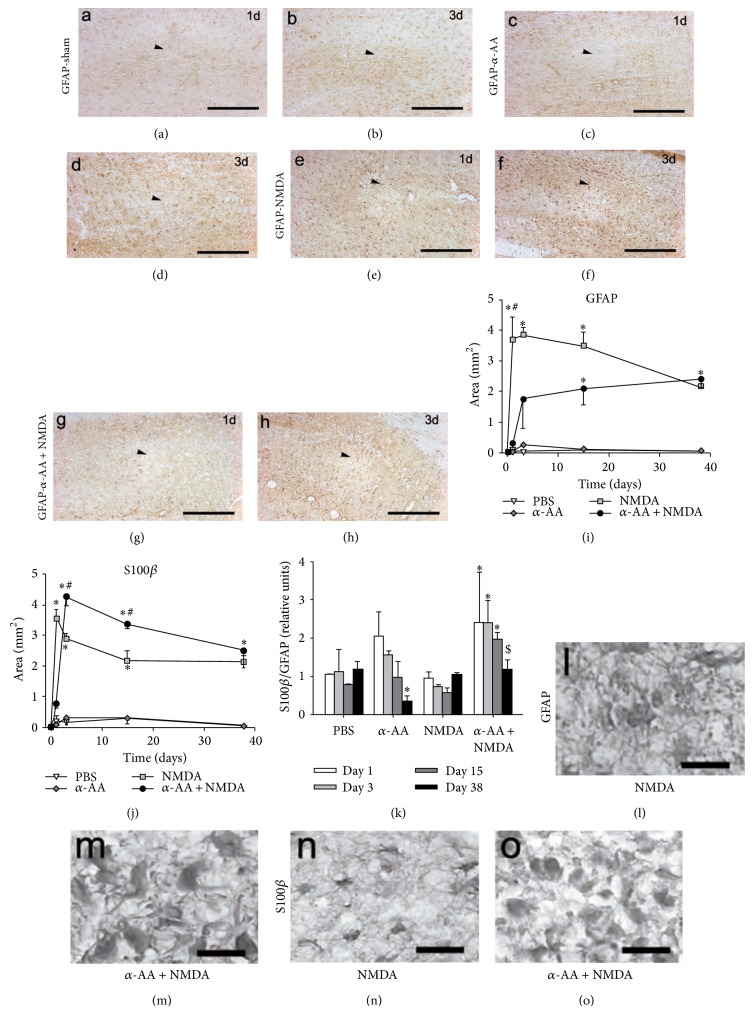
*α*-AA effect on the NMDA-induced astroglial reaction in the hippocampus. GFAP-immunostaining 1 (a) and 3 days (b) in sham animals, 1 (c) and 3 days (d) after *α*-AA, 1 (e) and 3 days (f) after NMDA lesion, and 1 (g) and 3 days (h) after *α*-AA + NMDA injection (arrowheads show the injection site). Please note in (c) and (g) the lack of GFAP-immunopositive cells in the surroundings of the injection site. Graphs show the quantification of the area of astrogliosis in the whole hippocampus. (i) GFAP-immunostained and S100B-immunostained (j) sections of sham (PBS), *α*-AA, NMDA, and *α*-AA + NMDA rats at postlesion days 1, 3, 15, and 38. (k) Graph shows the estimation of the ratio S100B/GFAP, calculated as the quotient between the area of increased S100B and the area of increased GFAP in these rats. Photomicrographs illustrate GFAP-immunoreactive cells of NMDA (l) and *α*-AA + NMDA (m) rats and S100B-immunoreactive cells of MNDA (n) and *α*-AA + NMDA (o) rats in the hippocampal parenchyma 3 days after the lesion. ^∗^ *P* < 0.05 different from PBS; ^#^ *P* < 0.05 different from NMDA; ^$^ *P* < 0.05 different from day 15 (LSD* post hoc* test) (*n* = 6 rats/group). Bar: 1 mm in (a)–(h) and 10 *μ*m in (l)–(o).

The treatment only with *α*-AA induced a loss in the GFAP-immunoreactivity (IR) in a small area of the hippocampus around the injection site that was already recovered at day 3 after injection (Figures 4(a)–4(d)). In the NMDA group, the GFAP-IR increase was maximal at day 13 (Figures 4(c) and 4(d)) and then progressively decreased by day 38. In the *α*-AA + NMDA group only a few astrocytes showed a weak reactive morphology at day 1 (Figures 4(e)–4(h)). At day 3, the area of enhanced GFAP-IR covered 29 ± 7% of the HF (Figure 4(i)), which increased slightly by day 38 to reach similar values to those obtained in the NMDA group at this time point. At day 38, reactive hippocampal astrocytes of *α*-AA + NMDA rats presented enhanced hypertrophy and hyperplasia and stronger GFAP-IR than did those of the NMDA group (Figures 4(l) and 4(m)).

Experiments of immunohistochemistry with S100B showed a very small area of increased IR that corresponded to the area of GFAP-IR loss, at day 1 in the *α*-AA group. In the NMDA rats, S100B-IR was maximal at day 1 and then progressively decreased by day 38, following the same dynamics as GFAP-IR did (Figures 4(j), 4(n), and 4(o)). In the *α*-AA + NMDA group the increased S100B-IR area was not different to sham group at day 1, and it reached a maximal value at day 3, before progressively decreasing to values similar to the NMDA group at day 38. When we calculated the ratio between S100B and GFAP (S100B/GFAP, Figure 4(k)) we found similar values between the sham and NMDA groups, whereas we found a significant increase in the *α*-AA + NMDA at days 3, 5, and 15 (Figure 4(k)). We found no expression of S100B in microglia using double immunohistochemistry and confocal analysis of anti-S100B with anti-CD11b antibodies (data not shown).

### 3.4. *α*-AA Increased NMDA-Induced Microgliosis and YM1 Production Three Days after the Lesion

**Figure 5:**
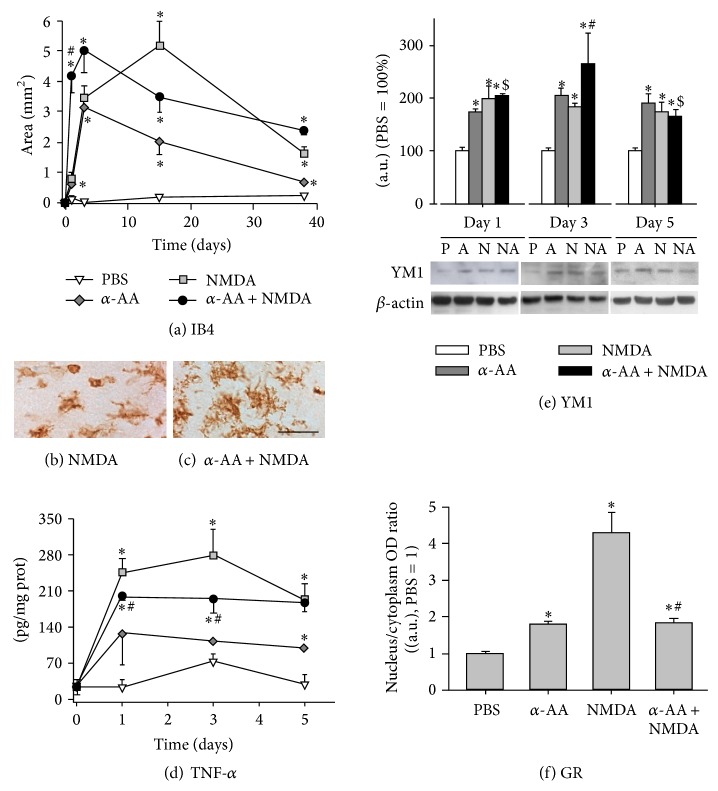
*α*-AA effect on the NMDA-induced microglial reaction in the hippocampus. (a) Quantification of the hippocampal area of microglial reaction in IB4-stained sections of sham (PBS), *α*-AA, NMDA, and *α*-AA + NMDA rats at postlesion days 1, 3, 15, and 38. Photomicrographs show hippocampal IB4-stained cells illustrating different microglial morphology between NMDA (b) and *α*-AA + NMDA (c) rats 3 days after the lesion. (d) TNF-*α* hippocampal concentration in PBS, *α*-AA, NMDA, and *α*-AA + NMDA rats during the first 5 days of the study. (e) Immunoblots and densitometric analysis (graphs) of YM1 in the hippocampus of PBS, *α*-AA, NMDA, and *α*-AA + NMDA rats during the first 5 days of the study. Values were normalized to *β*-actin bands. (f) Quantification of the nucleus/cytoplasm ratio of GR-immunolabeling in hippocampal glial cells 3 days after the lesion of the four groups of the study. P, PBS; A, *α*-AA; N, NMDA; NA, *α*-AA + NMDA; ^∗^ *P* < 0.05 different from PBS; ^#^ *P* < 0.05 different from NMDA; ^$^ *P* < 0.05 different from *α*-AA (LSD* post hoc* test in (a), (e); Student's *t*-test in (d), (f)) (*n* = 6 rats/group). Bar: 10 *μ*m.

In the *α*-AA group, the histochemistry with IB4 stained morphologically reactive microglia already at day 1 in an area that reached a maximal value at day 3, before decreasing to sham group values at day 38 (Figure 5(a)). In NMDA-lesioned group the area of microglial reactivity reached significance at day 1 and increased by day 15 and then decreased at day 38 (Figure 5(a)). In the *α*-AA + NMDA group, microglial reactivity was already significant at day 1 and covered a maximal area at day 3, to then progressively decrease at day 38 (Figure 5(a)). At days 1 and 3, reactive microglia of the *α*-AA + NMDA group presented a ramified morphology clearly different to the shape showed by microglial cells of the NMDA group, which presented clear reduction of processes (Figures 5(b) and 5(c)).

We assessed the TNF-*α* concentration in hippocampus by ELISA. In the HF of *α*-AA group, the TNF-*α* concentration was increased at days 1 and 5 when compared with sham group values (Figure 5(d)). In the NMDA group the TNF-*α* concentration in the HF increased at day 1 and it was maximal at day 3 and then it decreased at day 5. In the *α*-AA + NMDA group, TNF-*α* concentration was significantly lower than in the NMDA group (Figure 5(d)).

We assessed the YM1 concentration in hippocampus by Western blot. YM1 concentration was similarly enhanced in the *α*-AA and NMDA groups when compared with sham group values (Figure 5(e)). In the *α*-AA + NMDA group, the YM1 concentration was significantly higher compared with all groups for days 1 and 3, reaching a maximal increase at day 3 when compared with sham group (Figure 5(e)).

With respect to the nucleus/cytoplasm ratio of GR-IR in microglia, it was similarly increased in the *α*-AA and *α*-AA + NMDA groups when compared with sham group values at day 3, whereas this increase was higher in the NMDA group (Figure 5(f)).

## 4. Discussion

Brain NMDA microinjection triggers an excitotoxic process that expands through several dynamic processes resulting in astro- and microglial reactions and a massive neuronal loss [29] that may involve recruitment of peripheral neutrophils and macrophages [35]. The acute excitotoxic process is thought to be under control by retaliatory mechanisms within 15 days, but an underlying long-term process of at least 38 days goes on, associated with tissue atrophy, neuronal loss, and chronic microgliosis, in absence of astroglial reaction [32]. The microglia response may initially course with neuroprotective effects to afterwards cause neuron injury or death [2]. Thus, interventions that promote the regeneration of damaged tissue long after injury should take into consideration this dynamic phenomenon, in particular those addressed to interfere the neuroinflammatory pathways [7, 36, 37].

Although astrocytes present neuroprotective activity against excitotoxicity [24], we herein found that early astroglia ablation with *α*-AA does not enhance within the first 15 days the NMDA-induced hippocampal lesion. The injured hippocampal area in particular CA2 and CA3 subfields only increased at day 38. In parallel, *α*-AA increased the NMDA-induced microglia reaction from day 1 on. These results argue for an early potentiation of microglia neuroprotective activity while astrocytes are compromised [38].

Our results of IB4 histochemistry and TSPO labelling by* in vitro* autoradiography evidenced an early microglial activation that persisted at least for 38 days during which TNF-*α* secretion and YM1 and GR genes expression followed different patterns of activation. Specifically, the decrease of the initial massive production of TNF-*α* did not parallel the morphological long-term activation, YM1 secretion took place transiently during the first three days of the lesion, and GR translocation to the nucleus increased progressively up to day 5 before decreasing to control level. Since microglial phenotype results from the compromise between cytotoxic and trophic activities [1], these patterns result from the evolution of the microglial role during excitotoxicity.

The production of YM1 in the first five days of the lesion is related to a neuroprotective activity [9], as evidenced in the olfactory bulb axotomy that results in YM1 secretion that reduces the inflammatory process [39]. By contrast, TNF-*α* production and GR translocation to the nucleus reflect more complex roles of microglial activity. TNF-*α* secretion is crucial for autocrine fast microglial activation with cytotoxic effects, a process also fed with the concomitant release of TNF-*α* by reactive astrocytes [40, 41]. However, the absence of TNF-*α* in knockout mice delays NO-mediated microglial activation, resulting in further exacerbated microgliosis [20] that leads to amplification of secondary excitotoxicity. TNF-*α* can bind to two specific receptors: TNFR1, with an intracellular death domain, and TNFR2, with a higher affinity and mostly involved in neuroprotection [42]. Consequently, at low concentration TNF-*α* activates TNFR2-mediated neuroprotection, whereas at high concentration TNFR1 contributes to cell injury [43]. Similarly, at baseline concentration and as the immediate response to acute stress, glucocorticoids produce an anti-inflammatory activity over microglia [44]. However chronic high concentration of glucocorticoids exacerbates cytotoxic microgliosis and increases hippocampal injury due to excitotoxicity or ischemia [14]. Such a sustained elevated glucocorticoid concentration downregulates GR microglia expression as a prerequisite to specifically inhibit the anti-inflammatory actions [15]. Therefore, the early activation and posterior downregulation of microglial GR herein observed may enhance microglia cytotoxic and inflammatory activity.

S100B modifies astrocytic, neuronal, and microglial activities, and its effects depend on both its own extracellular concentration and the expression of the receptor RAGE. In activated microglia, S100B at micromolar concentrations upregulates interleukin-1*β* and TNF-*α* expression via RAGE [45], while at nanomolar concentrations S100B blocks that expression [18]. In turn, microglial reactive oxygen species, or TNF-*α*, modify the RAGE response to S100B [17], in a context of microglia-astroglia cross talk that integrates different signaling systems. In this regard, our results suggest that factors associated with hippocampal excitotoxic injury may trigger an early trophic neuroprotective reaction in microglia. Afterwards, variations in the concentration of these same factors may turn the chronic microgliosis into a proinflammatory cytotoxic activity.

The ablation of astroglia using *α*-AA results in a similar pattern of NMDA-induced hippocampal damage. *α*-AA did not modify the NMDA-induced hippocampal lesion area and neuronal loss during the first fifteen days, and we detected a discrete increase of these lesion parameters only in the CA2 and CA3 subfields at day 38, when astroglia was fully recovered. The *α*-AA-induced potentiation of microgliosis that we found in these conditions supports an early neuroprotective microglial activity [46]. As previously shown [24, 46], *α*-AA inhibits the main astroglial pathways of glutamate removal resulting in increased synaptic glutamate and increase of oxidative stress [23, 24, 26, 47], which constitute activation signals to microglia. This initial microglial phenotype may represent an attempt to preserve neurons during astroglial dysfunction. The activated microglia express glutamine synthetase and the glutamate transporter 1 in the early stages of excitotoxicity [47]. Microglial glutamate uptake starts at 4 h and lasts up to 72 h after the lesion [48], which indicates that reactive microglia may account for the control of synaptic glutamate homeostasis after early astrocyte injury. Furthermore, the early increase of YM1 concentration in the HF of *α*-AA + NMDA rats also suggests a neuroprotective microglial activity that may potentiate the recovery of astrocytes as indicates the slight enhancement of GFAP concentration herein found at day 3 in *α*-AA rats.

Initially the *α*-AA + NMDA lesion presented a decreased TNF-*α* concentration and a delay in the increase of S100B. Also, within the first three days, reactive microglia presented a morphological transition from a phagocytic to a ramified shape. Thus, when astrocytes are depleted, the retaliatory mechanisms against excitotoxicity would include the enhanced neurotrophic activity of the microglia to counteract the limited functional capacity of the regenerating astrocytes. Taken together, these data indicate a priority to recover the astrocyte-microglia cross talk before the astroglial cytoskeletal rearrangement and proliferation.

From day 3, our results in *α*-AA + NMDA rats showed a sustained reaction of microglia and a heightened S100B/GFAP ratio that resulted in an increased area of lesion that reached CA2 and CA3 hippocampal subfields at day 38 but no changes in the neuronal density of the lesioned CA1 subfields. These results also suggest that, rather than to the intensity of injury, the cytotoxic chronic activity of microglia contributes to the growth of affected areas during neurodegeneration. As the increased area of S100B-IR may not be translated into increased S100B release and could just reflect astrocytosis, further experiments are needed to quantify* in vivo* the S100B release long after an excitotoxic insult and characterize its effects on microglia inflammatory activity.

## 5. Conclusions

In conclusion, in this paper we show that NMDA-induced excitotoxicity in the hippocampus induces a chronic microgliosis. In this model, an initial release of YM1 followed by TNF-*α* production and the transient activation of GR may determine a putative functional switch of microglia. Although a role for infiltrated neutrophils and macrophages must not be ruled out, the astroglial removal in the early stages of the lesion may potentiate an initial neuroprotective role of microgliosis and reveals the S100B as a key modulator of microglia phenotype. These results also contribute to understanding the interplay between the trophic, neuroprotective, inflammatory, and cytotoxic functions of astrocytes and microglia during the course of brain injury. The fine control of these processes requires a dynamic understanding of their interactions to allow effective development of approaches to neuroprotection.

## Figures and Tables

**Table 1:** [^3^H]11195 and [^3^H]corticosterone specific binding to brain sections after 20 nmol NMDA injection in the hippocampus.

	0 days	1 day	5 days	15 days	38 days

Specific TSPO binding					
Sham hippocampus	481 ± 98	1532 ± 107^#^	618 ± 187	483 ± 341	483 ± 102
NMDA hippocampus	480 ± 73	1495 ± 189^*^	1644 ± 255^∗#^	1571 ± 346^∗#^	1440 ± 235^∗#^
Parietal cortex	490 ± 11	474 ± 64	472 ± 40	473 ± 18	488 ± 9
Piriform cortex	468 ± 5	439 ± 35	425 ± 33	453 ± 37	465 ± 2
Specific GR binding					
Sham hippocampus	68 ± 72	101 ± 94	74 ± 37	81 ± 13	75 ± 21
NMDA hippocampus	76 ± 62	198 ± 104	448 ± 247^∗#^	91 ± 23	105 ± 32
Parietal cortex	101 ± 19	102 ± 14	85 ± 19	77 ± 32	102 ± 20
Piriform cortex	103 ± 24	74 ± 19	89 ± 6	73 ± 3	102 ± 42

18 kDa translocator protein (TSPO) concentration was estimated by [^3^H]PK11195 specific binding. 5 *μ*M RU-28362 allowed discriminating the [^3^H]corticosterone specific binding to glucocorticoid receptors (GR). Data are expressed in fmol/mg of protein (mean ± S.E.M). ^*^ *p* < 0.05 different from 0 days; ^#^ *p* < 0.05 different from sham values (KW test).
